# The Role of PGK1 in Promoting Ischemia/Reperfusion Injury-Induced Microglial M1 Polarization and Inflammation by Regulating Glycolysis

**DOI:** 10.1007/s12017-023-08736-3

**Published:** 2023-02-07

**Authors:** Wei Cao, Zhengzhe Feng, Deyuan Zhu, Suya Li, Meng Du, Shifei Ye, Dayong Qi, Peng Li, Yan Chen, Yibin Fang

**Affiliations:** 1 Department of Neurovascular Disease, School of Medicine, Shanghai Fourth People’s Hospital, Tongji University, 1279 Sanmen Road, Shanghai, 200080 China; 2 Neurovascular Center, Changhai Hospital, Naval Medical University, Shanghai, China; 3 Department of Neurosurgery, The Second Affiliated Hospital Zhejiang University School of Medicine, 88 Jiefang Road, Hangzhou, 310009 China

**Keywords:** Stroke, Microglia cells, Inflammation, Glycolysis, Acetylation

## Abstract

Stroke is a leading cause of death, with a continuously increasing incidence. As a metabolic process that catabolizes glucose pyruvate and provides adenosine triphosphate (ATP), glycolysis plays a crucial role in different diseases. Phosphoglycerate kinase 1 (PGK1) facilitates energy production with biosynthesis in many diseases, including stroke. However, the exact role of PGK1/glycolysis in stroke remains to be elucidated. A rat model of middle cerebral artery occlusion (MCAO) was used to mimic ischemia/reperfusion injuries. Oxygen glucose deprivation/re-oxygenation (OGD/R) was used to induce injury to highly aggressively proliferating immortalized (HAPI) rat microglial cells. The extracellular acidification rate (ECAR) was determined using an XFe24 Extracellular Flux Analyzer. ATP, lactate dehydrogenase, tumor necrosis factor-alpha, and interleukin-6 levels were measured using commercial kits. Chromatin immunoprecipitation assay was performed to examine the interaction between H3K27ac or p300 and the PGK1 promoter region. PGK1 was either knocked down or overexpressed by lentivirus. Thus, to examine its role in stroke, real-time polymerase chain reaction and immunoblotting were used to measure gene expression. The expression of PGK1 was increased and associated with M1 polarization and glycolysis in MCAO rat models. OGD/R promoted M1 polarization and HAPI microglial cell inflammation by regulating glycolysis. Silencing PGK1 reduced OGD/R-increased M1 polarization, inflammation, and glycolysis. Conversely, the overexpression of PGK1 promoted HAPI microglial cell inflammation by regulating glycolysis. The mechanism showed that histone acetyltransferase p300 promoted PGK1 expression through H3K27 acetylation. Finally, data indicated that silencing PGK1 inhibited microglia M1 polarization, inflammation, and glycolysis in MCAO rat models. PGK1 could promote ischemia/reperfusion injury-induced microglial M1 polarization and inflammation by regulating glycolysis, which might provide a novel direction in developing new therapeutic medications for preventing or treating stroke.

**Supplementary Information:**

The online version contains supplementary material available at 10.1007/s12017-023-08736-3.

## Introduction

Stroke is a common disease and a leading cause of death worldwide (Collaborators et al., 2018). It is classically characterized as a neurological deficit because of acute central nervous system (CNS) injury by a vascular cause, such as cerebral infarction and intracerebral hemorrhage (Sacco et al., 2013). Its common symptoms include headache, aphasia, and hemiparesis (Fekadu et al., 2019). High cholesterol levels, diabetes mellitus, smoking, and the lack of physical activity are risk factors (Boehme et al., 2017). Microglia are the immune cells of the CNS and are critically involved in brain infections and inflammation (Wake et al., 2011). Stroke activates microglia and downstream signaling (Hu et al., 2012). Meanwhile, M1 microglia increase secondary brain damage, whereas M2 microglia improve recovery after stroke (Jiang et al., 2020). Better understanding the pathogenesis mechanism by which microglia regulate brain damage will help prevent and treat stroke.

Glycolysis catabolizes glucose to pyruvate and provides adenosine triphosphate (ATP) (Pelicano et al., 2006). Many other metabolic pathways, including fatty acid synthesis, nucleotide synthesis, and gluconeogenesis, are strongly reliant on glycolysis (Li et al., 2015). Studies reported that glycolysis plays a significant role in neurological diseases. For instance, Sonntag et al*.* have reported that glucose uptake and the glycolytic rate were sharply increased in Alzheimer’s disease (Sonntag et al., 2017). More importantly, enhancing astrocytic glycolysis has been proposed to promote neuronal protection during acute ischemic stroke (Takahashi, 2021). Hyperglycolysis is also observed in stroke and ischemia (Cheng et al., 2021). Regardless of the advances in the study of glycolysis, the role of glycolysis in stroke remains unclear.

Phosphoglycerate kinase 1 (PGK1) is an essential enzyme that catalyzes the formation of ATP in the aerobic glycolysis pathway (He et al., 2019). PGK1 deficiency has been associated with parkinsonism, hereditary non-spherocytic hemolytic anemia, neurological impairment, and myopathy (Beutler, 2007; He et al., 2019). Extracellular PGK1 benefits the survival of dopaminergic neurons and decreases neurotoxin damage to alleviate Parkinson’s disease (Lin et al., 2022). PGK1 downregulation prevents traumatic brain injury by inhibiting oxidative stress by activating NRF2 signaling (Xu et al., 2022). Chen et al*.* showed that the activation of PGK1 with terazosin suppressed organ damage and increased the survival of animals with stroke (Chen et al., 2015). It has long been shown that both enhanced PGK1 activity and increased glycolysis alleviate neurodegeneration (Cai et al., 2019) and that energy deficiency may be a pathogenic factor for the pathogenesis of neurodegenerative diseases, leading to neuronal dysfunction and subsequent death (Saxena, 2012). However, the roles of PGK1 in glycolysis and stroke remain to be elucidated.

## Materials and Methods

### Construction of an Ischemia/Reperfusion Model

Eighteen Sprague–Dawley rats (7–8 weeks old) (Guangdong Medical Lab., Guangzhou) were used to establish MCAO rat models, which were randomly divided into sham (control), MCAO + shNC, and MCAO + shRNA-Pgk1 (MCAO + shPgk1) groups (*n* = 6). shNC or shPgk1 was administered to the right lateral ventricle using a syringe. After anesthetization, a ventral midline incision was made to expose the carotid artery. Then, a suture (0.26 mm) was inserted into the right common carotid artery lumen and advanced into the internal carotid artery up to 18 mm distal to the bifurcation. The suture was removed 90 min after achieving reperfusion, and then, the incisions were closed. For controls, an incision was made to expose the three aforementioned arteries and then closed. Rats in the MCAO + shNC or MCAO-shRNA-Pgk1 group were administered with shNC or shPgk1 15 min before establishing MCAO, respectively (Zhang et al., 2018). The rats were euthanized after 14 days, and brain tissues were collected.

### Neurological Score

After 24 h of MCAO model reperfusion, the neurological scores were evaluated following the following scoring system: score 0, no neurological deflect; score 1, failure to expand contralateral forepaw fully; score 2, circling contralateral side; score 3, falling toward contralateral side; and score 4, did not walk instinctively.

### Single-Cell RNA Sequencing

The rat brain was mechanically disrupted with a phosphate-buffered solution and soaked in 4-mL papain/DNAse for 25 min at 37 °C, triturated, strained, and washed with Hanks’ Balanced Salt Solution. Single-cell suspension was loaded onto a chromium platform using a C gel bead kit v3 (10X Genomics, Pleasanton, CA), to capture 6000 cells per library. Libraries were sequenced using a NovaSeq 6000 System (Illumina). Then, downstream analysis was performed using Scanpy (V1.7.1) in Python. Dimension reduction started with PCA. A neighborhood graph was made, and the best-matched *k*-Nearest Neighbor was automatically weighted. Then, clustering analysis was performed, and the main cell types were defined using the SingleR (Aran et al., 2019), followed by differential expression analysis between controls and MCAO. Then, we identified the expression of PGK1 between microglial cells from the controls and those from MCAO rat models. Differentially expressed genes between treatments were calculated using the Wilcoxon rank-sum test. The Molecular Signatures Database (MSigDB) was used for signature calculation.

### Cell Culture and Treatment

HAPI cells from Enzyme-Linked Bio. (Shanghai) were cultured in Dulbecco’s Modified Eagle Medium with 10% fetal bovine serum (Sigma, Shanghai) at 37 °C. The cells were cultured in a glucose-free medium and an atmosphere with 95% N_2_ and 5% CO_2_ or 5% CO_2_ for 4 h. The normal medium was provided for 4 h after oxygen and glucose deprivation (OGD), and the cells were cultured for 3, 6, 12, or 24 h with/without the glycolysis inhibitor, 2-DG (25 μM).

### Cell Transfection

The pLVX-Puro plasmid was used to construct the Pgk1 overexpression vector: pLKO.1-Pgk1-shRNA (shPgk1-1, shPgk1-2, and shPgk1-3) and pLKO.1-p300-shRNA (shp300-1, shp300-2, and shp300-3) were used to construct Pgk1 and p300 knockdown models, respectively. Blank control plasmid (vector), pLVX-Puro-Pgk1 (oePgk1), pLKO.1-Pgk1-shRNA, pLKO.1-p300-shRNA, and pLKO.1-scramble-shRNA (shNC) was transfected into 293 T cells using Lipo2000 (Invitrogen). Lentivirus was collected after 48 h and used for infecting HAPI microglia.

### Enzyme-Linked Immunosorbent Assay (ELISA)

Tumor necrosis factor-alpha (TNF-α) and interleukin-6 (IL-6) levels in the cell medium were measured using commercial kits: TNF-α (Thermo-Fisher Scientific, 88-7340-22) and IL-6 (Abcam, ab234570). Myeloperoxidase (MPO) activity in brain tissues was measured using Rat Myeloperoxidase ELISA kit (Abcam, ab285308).

### Extracellular Flux (XF) Analysis

Glycolysis levels were monitored by measuring the ECAR, as previously mentioned (Guo et al., 2020).

### Lactate Dehydrogenase (LDH) Levels

LDH levels in cells were detected using commercial kits (Biovision).

### Quantitative Real-Time Polymerase Chain Reaction (qPCR)

RNAs were extracted from HAPI cells with TRIzol, quantified, and reverse transcribed into cDNA for qPCR assay using SYBR Green Master Mix (Roche) on an ABI 7500 cycler. The results were calculated using the 2^−ΔΔCt^ formula. The primers were (5′–3′) PGK1: CGGAGACACCGCCACTTG (F), CCCGATGCAGTAAAGACGAG (R); iNOS: TTGGAGCGAGTTGTGGATTG (F), GGAACGTGGGGTTGTTGC (R); IL-1β: TATGTCTTGCCCGTGGAGC (F), CACAGGGATTTTGTCGTTGC (R); p300: TGCAAGGGATAAGCACCTGG (F), GCTTGCTGGTTGTTGCTCTC (R); and GAPDH: CAACGACCCCTTCATTGACC (F), CACCCCATTTGATGTTAGCG-3′ (R).

### Immunoblotting

Proteins were extracted, quantified, resolved in sodium dodecyl sulfate–polyacrylamide gel electrophoresis, and electroblotted to polyvinylidene fluoride membranes. The membranes were probed with antibodies against PGK1 (CST, 68540S), PKM2 (CST, 4053T), LDHA (CST, 3582T), H3K27ac (CST, 8173T), p300 (CST, 54062S), GAPDH (Proteintech; 60004-1-Ig), and secondary antibodies (Beyotime, A0208).

### Plasmid Construction and Luciferase Assay

The cells were transfected with PGL3-promoter plasmid harboring the 5′-promoter region of Pgk1 and pRL-TK plasmid using Lipo2000 with C646 (20 μM) for 6 h. Then, luciferase activity was measured.

### Chromatin Immunoprecipitation (ChIP)

ChIP assay was performed, as previously mentioned (Zhu et al., 2017), using antibodies against H3K27ac, p300, or control IgG. Purified ChIP DNA was confirmed using PCR. Pgk1 primer sequences were as follows: 5′-GCCACCTTCTACTCCTCCCC-3′ (forward), 5′-TGCCCGATTCCATTGCTC-3′ (reverse).

### Statistical Analysis

Data are presented as means ± standard deviations and analyzed using Prism 8.4.2. Comparisons between two groups were performed using Student’s *t *test, and multiple comparisons were performed using one-way analysis of variance. *P *values of less than 0.05 were used to indicate statistical significance.

## Results

### Increased PGK1 was Associated with M1 Polarization and Glycolysis in the MCAO Rat Model

**Fig. 1:**
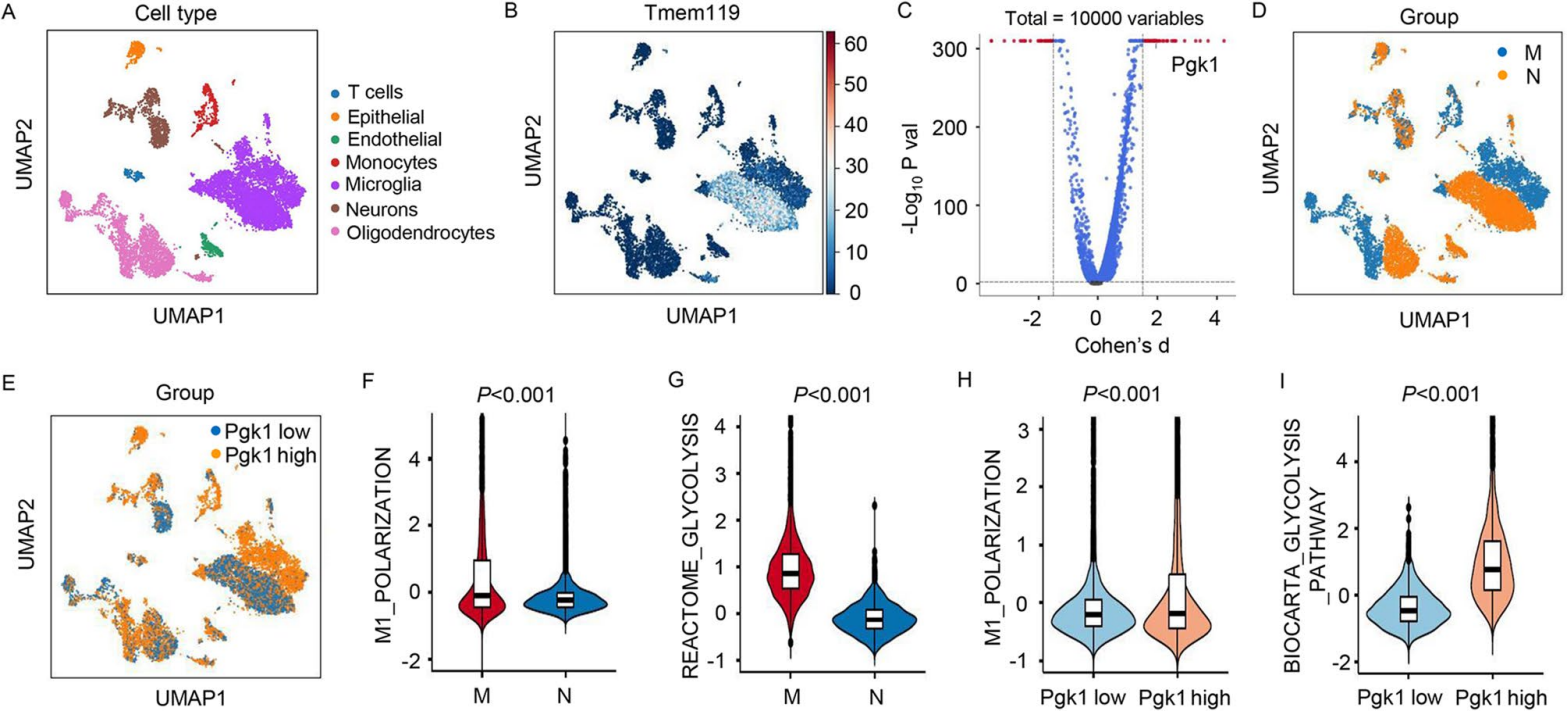
Increased PGK1 expression was associated with M1 polarization and glycolysis in the MCAO rat model. **A** The UMAP plot of all cells colored by each cell type in the MCAO rat model. **B** The UMAP plot showed the expression of microglial marker genes for microglial cells. **C** The volcano plots showed differentially expressed genes in microglial cells of rats with MCAO or normal rats. **D, E** The UMAP plot displayed the expression of PGK1 in different cells. The violin plot showed **F** M1_POLARIZATION and **G** REACTOME_GLYCOLYSIS in microglial cells of rats with MCAO or normal rats. The violin plot showed **H** M1_POLARIZATION and **I** BIOCARTA_GLYCOLYSIS_PATHWAY in microglial cells with high or low PGK1 expression levels. *M* MCAO, *N* normal

The MCAO rat model was used to assess the role of PGK1. First, different types of cells involved in MCAO were displayed in UMAP plots (Fig. 1A). Then, the expression of microglial marker genes for microglial cells was also displayed in UMAP plots (Fig. 1B). The differentially expressed genes in microglial cells shown by the volcano plot indicated that PGK1 was significantly upregulated in rats with MCAO compared with that in controls (Fig. 1C), and the expression levels of PGK1 in different cells were also displayed in UMAP plots (Fig. 1D, E). M1 polarization- and glycolysis-related MSigDB gene sets were downloaded and used to verify which gene sets had significant differences in microglial cells between rats with MCAO (M) and normal (N). The results demonstrated two significantly enriched gene sets, with normalized *P* values (< 0.05), from the following pathways: M1_POLARIZATION and REACTOME_GLYCOLYSIS (Fig. 1F, G). Moreover, we also used the aforementioned data to verify which gene sets had significant differences between microglial cells with high and low PGK1 expression levels. The results demonstrated two significantly enriched gene sets, with normalized *P* values (< 0.05), from the following pathways: M1_POLARIZATION and BIOCARTA_GLYCOLYSIS_PATHWAY (Fig. 1H, I). These findings suggest that increased PGK1 was associated with M1 polarization and glycolysis in the MCAO rat model.

### OGD/R Promoted HAPI Microglial Cell M1 Polarization and Inflammation by Regulating Glycolysis

**Fig. 2:**
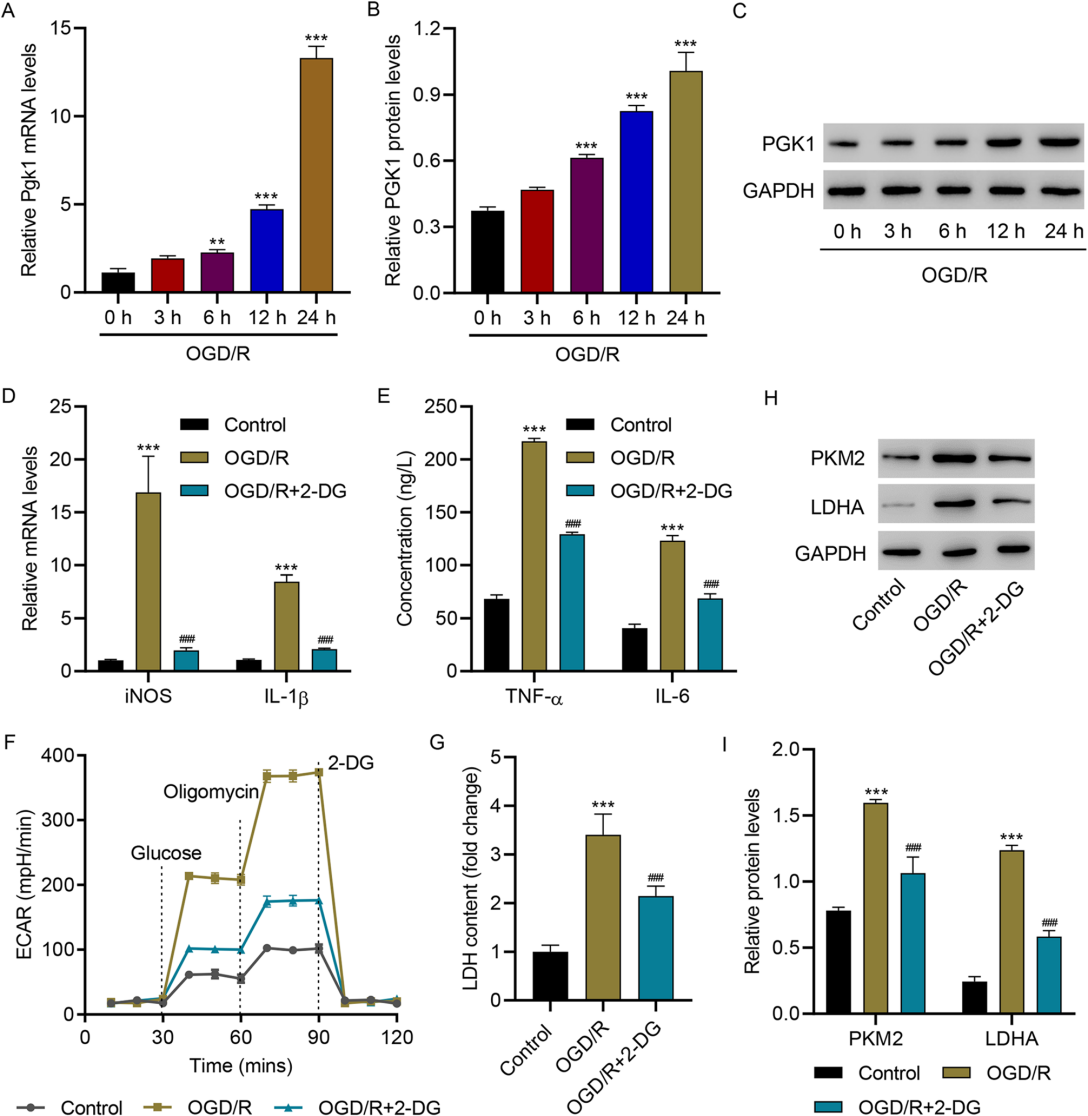
OGD/R promoted HAPI microglial cell M1 polarization and inflammation by regulating glycolysis. **A–C** PGK1 levels in HAPI cells 0, 3, 6, 12, and 24 h after OGD/R. **D** The mRNA levels of iNOS and IL-1β, **E** concentration of TNF-α and IL-6, **F** ECAR level, **G** LDH content, and **H, I** protein levels of PKM2 and LDHA in HAPI microglial cells 24 h after OGD/R. ***p < 0.001 vs. control; ^###^*p* < 0.001 vs. OGD/R

Next, an OGD/R in vitro model was introduced using HAPI microglial cells. After 0, 3, 6, 12, and 24 h, OGD/R time-dependently increased PGK1 levels (Fig. 2A–C). The data also indicated that OGD/R increased the mRNA levels of inducible nitric oxide synthase (iNOS) and IL-1β, which were reduced by inhibiting glycolysis with 2-DG (Fig. 2D). The levels of TNF-α and IL-6 were significantly upregulated by OGD/R but downregulated by 2-DG (Fig. 2E). OGD/R also significantly increased the ECAR (Fig. 2F) and LDH levels (Fig. 2G), which were reduced by 2-DG. Immunoblotting results showed that OGD/R remarkably enhanced the expression of PKM2 and LDHA in HAPI microglial cells, which was reduced by 2-DG (Fig. 1H, I). Collectively, these findings demonstrate that OGD/R promotes HAPI microglial M1 polarization and inflammation by regulating glycolysis.

### PGK1 Knockdown Inhibited OGD/R-Induced M1 Polarization, Inflammation, and Glycolysis

**Fig. 3:**
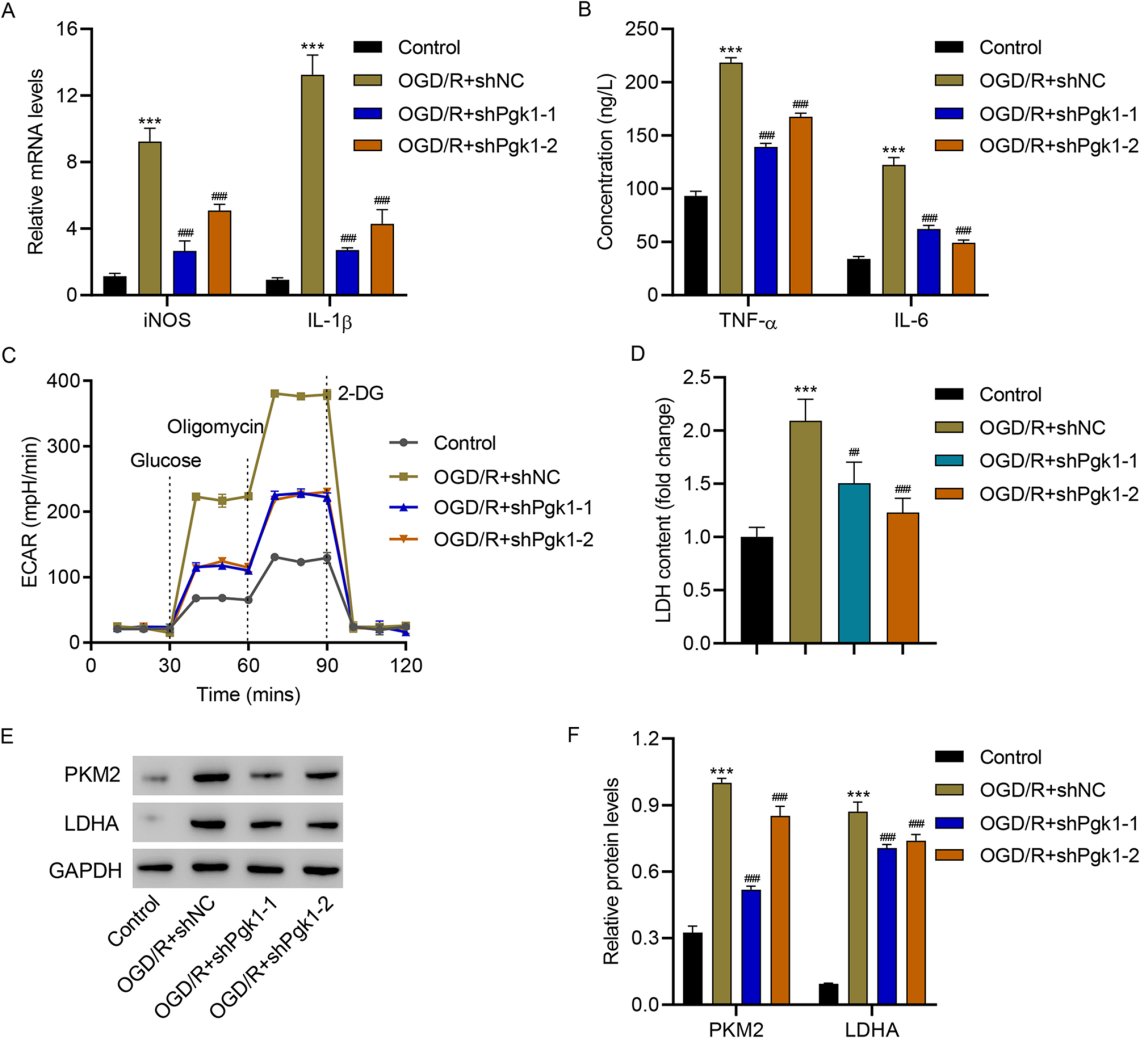
PGK1 knockdown inhibited OGD/R-induced HAPI microglial cell M1 polarization, inflammation, and glycolysis. **A** mRNA levels of iNOS and IL-1β, **B** TNF-α and IL-6 concentrations, **C** ECAR level, **D** LDH content, and **E, F** protein levels of PKM2 and LDHA in HAPI cells transduced with PGK1 shRNA or shNC 24 h after OGD/R. ***p < 0.001 vs. control; ^##^*p* < 0.01, ^###^*p* < 0.001 vs. OGD/R + shNC

Then, PGK1 was successfully silenced (Fig. S1) to further investigate its role. PGK1 silencing significantly reduced OGD/R-increased mRNA levels of iNOS and IL-1β (Fig. 3A), diminished OGD/R-increased levels of TNF-α and IL-6 (Fig. 3B), eliminated OGD/R-increased ECAR (Fig. 3C) and LDH levels (Fig. 3D), and reduced OGD/R-increased levels of PKM2 and LDHA (Fig. 1E, F). These results reveal that PGK1 silencing inhibits OGD/R-induced HAPI microglial cell M1 polarization, inflammation, and glycolysis.

### PGK1 Overexpression Promoted HAPI Microglial Cell Inflammation by Regulating Glycolysis

**Fig. 4:**
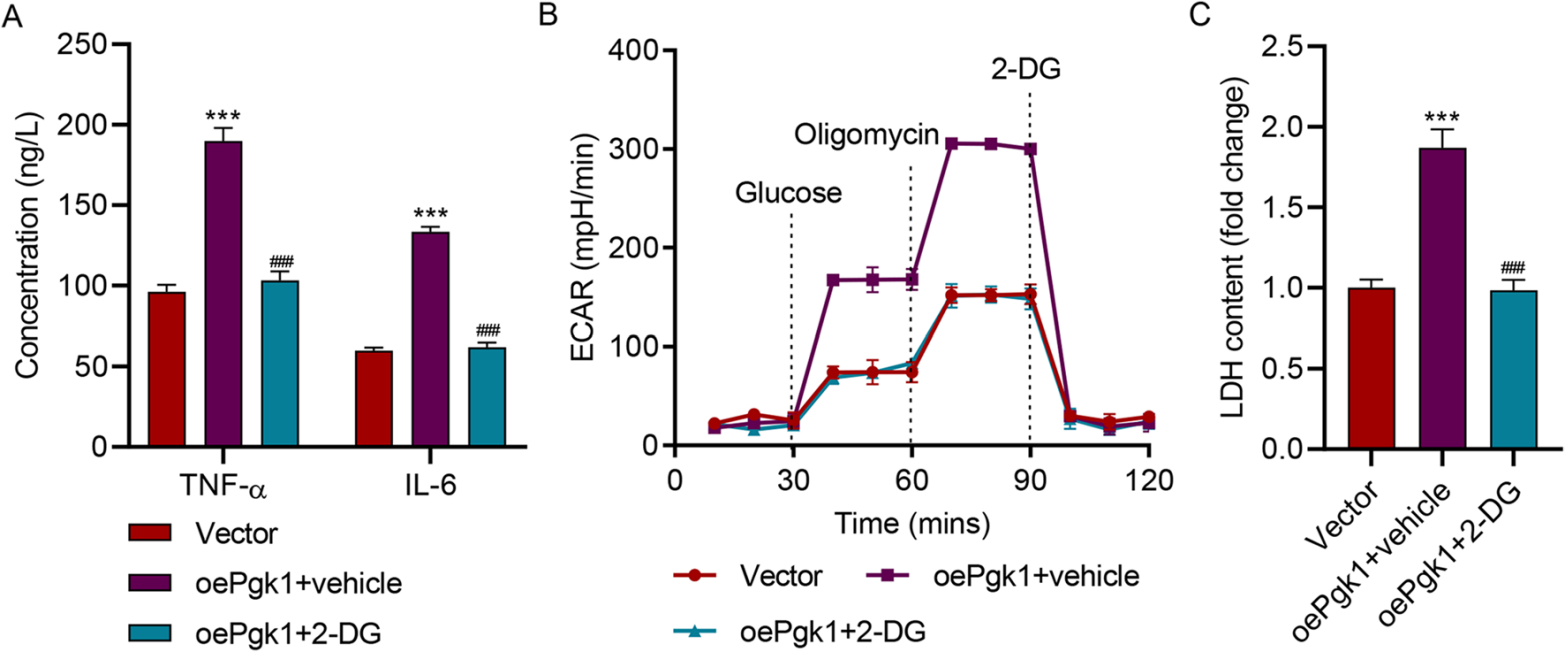
PGK1 overexpression promoted HAPI microglial cell inflammation by regulating glycolysis. **A** TNF-α and IL-6 concentrations, **B** ECAR level, and **C** LDH content in HAPI microglial cells transduced with the PGK1 expression vector and treated with 2-DG for 24 h. ***p < 0.001 vs. vector; ^###^*p* < 0.001 vs. oePgk1 + vehicle

PGK1 was also successfully overexpressed (Fig. S1) to further explore its role. Overexpressing PGK1 also significantly increased TNF-α and IL-6 (Fig. 4A), ECAR (Fig. 4B), and LDH levels (Fig. 4C), which were all reduced by 2-DG. These findings suggest that overexpressing PGK1 promoted HAPI microglial cell inflammation by regulating glycolysis.

### Histone Acetyltransferase p300 Promotes PGK1 Transcription Through H3K27 Acetylation

**Fig. 5:**
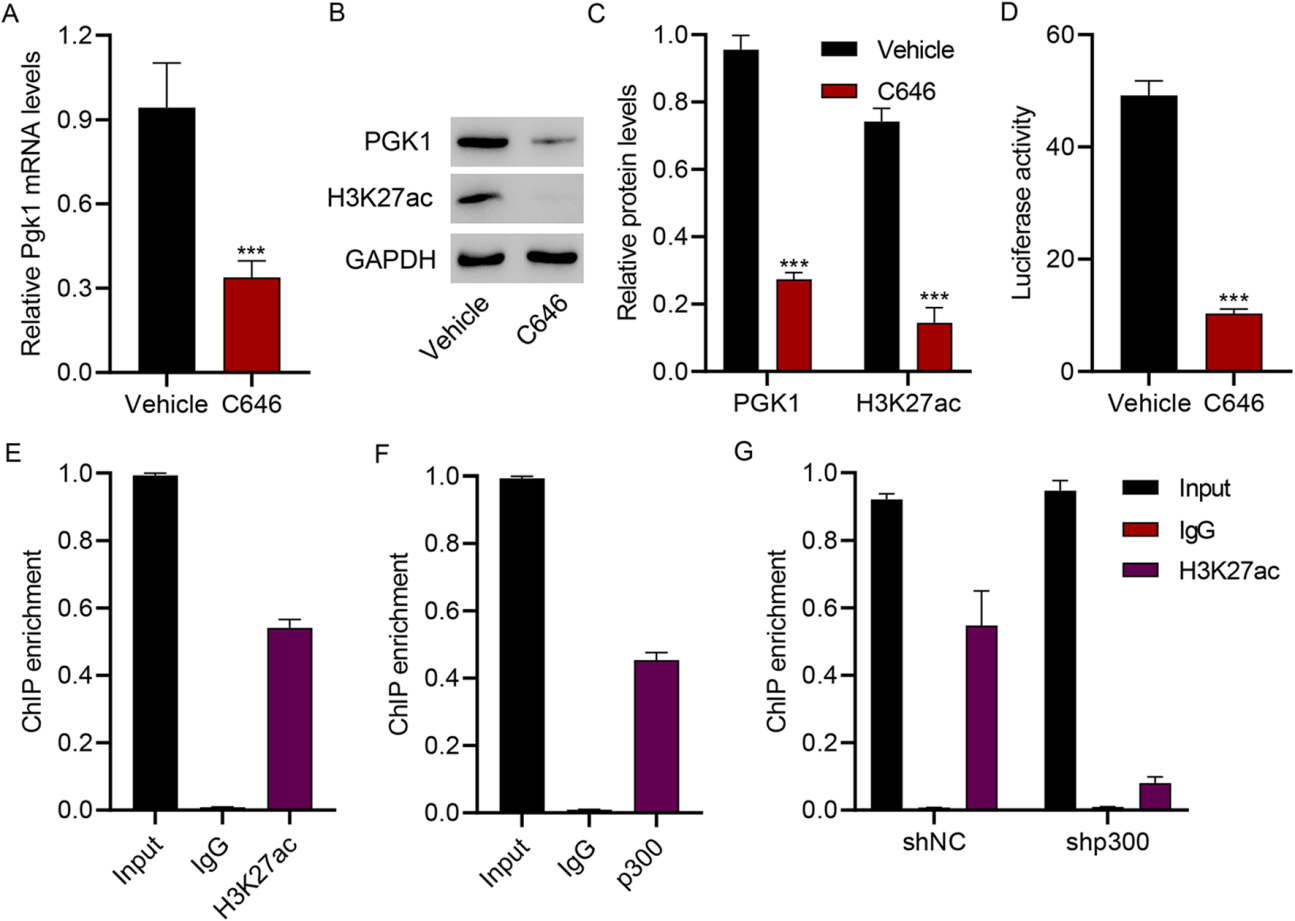
Histone acetyltransferase p300 promotes PGK1 transcription through H3K27 acetylation. HAPI microglial cells were treated with 10 μM histone acetyltransferase p300 inhibitor (C646) or its vehicle DMSO. **A–C** PGK1 expression and H3K27 acetylation (H3K27ac). **D** Luciferase activity of the PGK1 promoter. **E, F** ChIP assay of H3K27ac and p300 on the PGK1 promoter in HAPI cells. **G** ChIP assay of H3K27ac on the PGK1 promoter in HAPI cells transduced with p300 shRNA. ***p < 0.001 vs. vehicle

To understand PGK1 regulation, HAPI microglial cells were treated with histone acetyltransferase p300 inhibitor (C646) (10 μM). The administration of C646 significantly suppressed the expression of PGK1 and H3K27ac (Fig. 5A–C). Furthermore, C646 markedly inhibited PGK1 promoter activity (Fig. 5D). ChIP assay results indicated an interaction between H3K27ac and the PGK1 promoter (Fig. 5E) or between p300 and the PGK1 promoter (Fig. 5F). More importantly, data showed that p300 silencing (Fig. S1) significantly inhibited the interaction between H3K27ac and PGK1 promoter (Fig. 5G). Collectively, these findings demonstrate that histone acetyltransferase p300 increases PGK1 by regulating H3K27 acetylation.

### Silencing PGK1 Inhibited Microglia M1 Polarization, Inflammation, and Glycolysis in Rats with MCAO

**Fig. 6:**
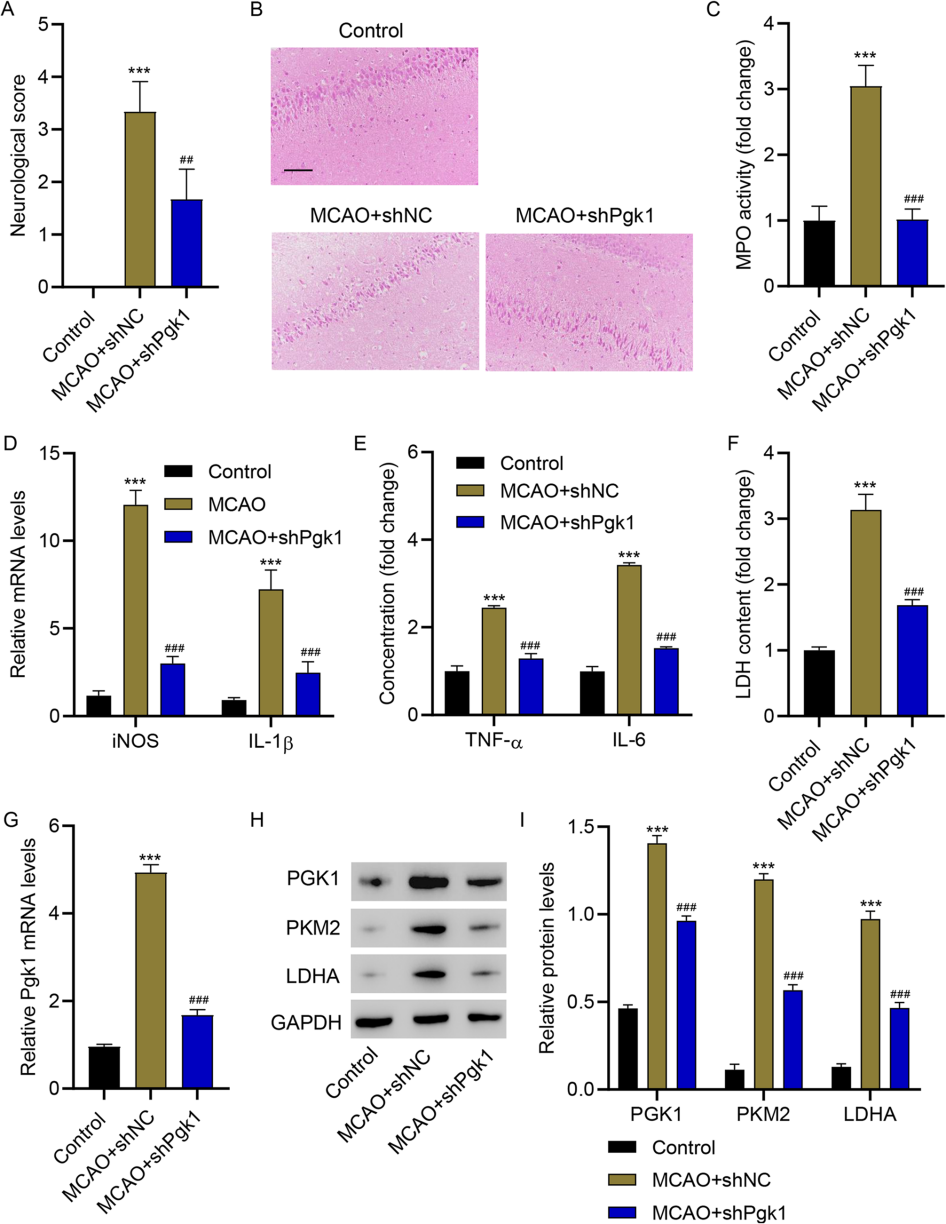
PGK1 silencing inhibited microglia M1 polarization, inflammation, and glycolysis in rats with MCAO. **A** Neurological score, **B** hematoxylin and eosin staining, **C** MPO activity, **D** mRNA levels of iNOS and IL-1β, **E** TNF-α and IL-6 concentrations, **F** LDH content, **G** PGK1 mRNA levels, and **H, I** PGK1, PKM2, and LDHA protein levels in brain tissues from rats with MCAO. ***p < 0.001 vs. control; ^##^*p* < 0.01, ^###^*p* < 0.001 vs. MCAO + shNC

Next, the effects of PGK1 silencing were investigated in vivo. Figure 6A shows the neurological score of the different groups of rats. Rats with MCAO demonstrated increased neurological scores. PGK1 silencing significantly reduced the neurological parameters. Hematoxylin and eosin staining of brain sections demonstrated that PGK1 silencing significantly decreased inflammatory cell infiltration (Fig. 6B). Furthermore, PGK1 silencing significantly inhibited MCAO-increased MPO activity (Fig. 6C); levels of iNOS and IL-1β (Fig. 6D), TNF-α and IL-6 (Fig. 6E), and LDH (Fig. 6F); and the expression of PGK1, PKM2, and LDHA (Fig. 1G–I) in the brain tissues of rats with MCAO. These results indicate that PGK1 silencing inhibits microglia M1 polarization, inflammation, and glycolysis in rats with MCAO.

## Discussion

This study demonstrated that PGK1 upregulation promoted M1 polarization and glycolysis in both the MCAO rat model and HAPI microglial cells. PGK1 silencing eliminated the effects of OGD/R on M1 polarization and glycolysis. Data also supported that the expression of PGK1 was promoted by histone acetyltransferase p300 through H3K27 acetylation. For the first time, this study indicates that p300-mediated PGK1 upregulation promoted M1 polarization and glycolysis through H3K27 acetylation, which may provide novel directions for drug development.

As mentioned above, stroke is a leading cause of death, and ischemic stroke accounts for approximately 70% of all stroke cases worldwide (Xue et al., 2021). As CNS parenchymal macrophages (Aguzzi et al., 2013), microglia could present different phenotypes, a process known as microglial polarization, including both M1 (proinflammatory) and M2 (anti-inflammatory) phenotypes (Girard et al., 2013). Microglial stimulation has been known to activate inflammatory transcription factors (Combs et al., 2001). Moreover, inflammation has been shown to contribute to ischemic stroke and ischemic brain injury (Jin et al., 2010). Stroke induces a systemic inflammation that triggers sickness behaviors (Wen et al., 2018). For example, Hou et al*.* reported that prolonged inflammation is correlated with poor prognosis in massive stroke (Hou et al., 2021). Our results indicated that PGK1 overexpression promoted HAPI microglial cell M1 polarization and inflammation. Conversely, PGK1 silencing eliminated the effects of OGD/R on M1 polarization and glycolysis both in vivo and in vitro. These results demonstrate that PGK1 plays a key role in regulating microglial cell M1 polarization and inflammation and improving the knowledge on PGK1 in stroke.

Glycolysis, which occurs in the cytoplasm, anaerobically converts glucose into pyruvate and provides ATP (Kierans & Taylor, 2021). Glucose metabolism provides greater energy for macrophages (Zhu et al., 2014). Then, glycolysis promotes a faster rate of ATP production in microglial cells (Lauro & Limatola, 2020). Wang et al*.* indicated that GLUT1 promotes glucose uptake in microglia, suggesting that microglial activation is critically controlled by facilitating glycolysis (Wang et al., 2019). Glycolysis orchestrates the behavior of inflammatory cells. Inflammation reprogrammed microglia toward glycolysis (Soto-Heredero et al., 2020). More importantly, inhibiting glucose metabolism and its associated hyperglycolysis has been reported to enhance the efficiency of hypothermia-induced neuroprotection in ischemic stroke (Han et al., 2021). In this study, PGK1 was found to critically regulate microglial cell M1 polarization and inflammation by regulating the expression of PKM2 and LDHA, which ultimately regulate glycolysis, highlighting the importance of glycolysis in microglial function.

Histone acetylation regulates the expression of genes to influence biological or pathological processes (Zhou et al., 2019). Acetylation is a modification that changes the function of a protein (Christensen et al., 2019). Dysregulated acetylation can cause different diseases, such as leukemia and epithelial cancers (Timmermann et al., 2001). A study demonstrated that H3K27ac is closely related to the epigenetic regulation of gene expression (Lavarone et al., 2019). Ma and Zheng demonstrated that lncRNA PAXIP1-AS1 activated by H3K27ac promotes ovarian cancer (Ma & Zheng, 2021). Disruption of BRD4 at the H3K27Ac-enriched enhancer region suppressed the expression of c-Myc in Merkel cell carcinoma (Sengupta et al., 2015). H3K27ac also contributes to inflammation and stroke. For example, the H3K27ac increase in special typical enhancers is associated with the development of intestinal inflammation (Chen et al., 2019). Another study revealed that restoring hyper-H3K27ac status suppressed the expression of overactivated brain inflammation-related genes (Cheng et al., 2018). Our results suggested that histone acetyltransferase p300 promoted PGK1 transcription, which inhibits microglial cell M1 polarization and inflammation. The mechanism of the study reveals that p300 promoted PGK1 transcription through H3K27 acetylation. These findings indicated the significance of the p300/H3K27ac/PGK1 axis in microglial cell growth and inflammation, which may benefit the study and treatment of stroke. Remarkably, only animals or animal cells were included in this study. Future studies with clinical samples can provide more useful information. Nevertheless, the study demonstrated new roles of PGK1 and glycolysis in stroke.

In conclusion, PGK1 upregulation by the p300/H3K27ac pathway promoted oxygen glucose deprivation-induced microglial cell M1 polarization and inflammation by regulating glycolysis, providing a novel direction in developing new therapeutic medications for the prevention or stroke.

## Supplementary Information

Below is the link to the electronic supplementary material.

Supplementary file1 (DOCX 394 KB)

## Data Availability

The data used to support the findings of this study are included within the article.
