# Supplementary material for: The Role of PGK1 in Promoting Ischemia/Reperfusion Injury-Induced Microglial M1 Polarization and Inflammation by Regulating Glycolysis
Source: Neuromolecular Med. 2023 Feb 7;25(2):301–11. doi: 10.1007/s12017-023-08736-3 (PMC10267262; doi:10.1007/s12017-023-08736-3)
Supplement: Supplementary file 1 — Supplementary file1 (DOCX 394 KB) [file 12017_2023_8736_MOESM1_ESM.docx]

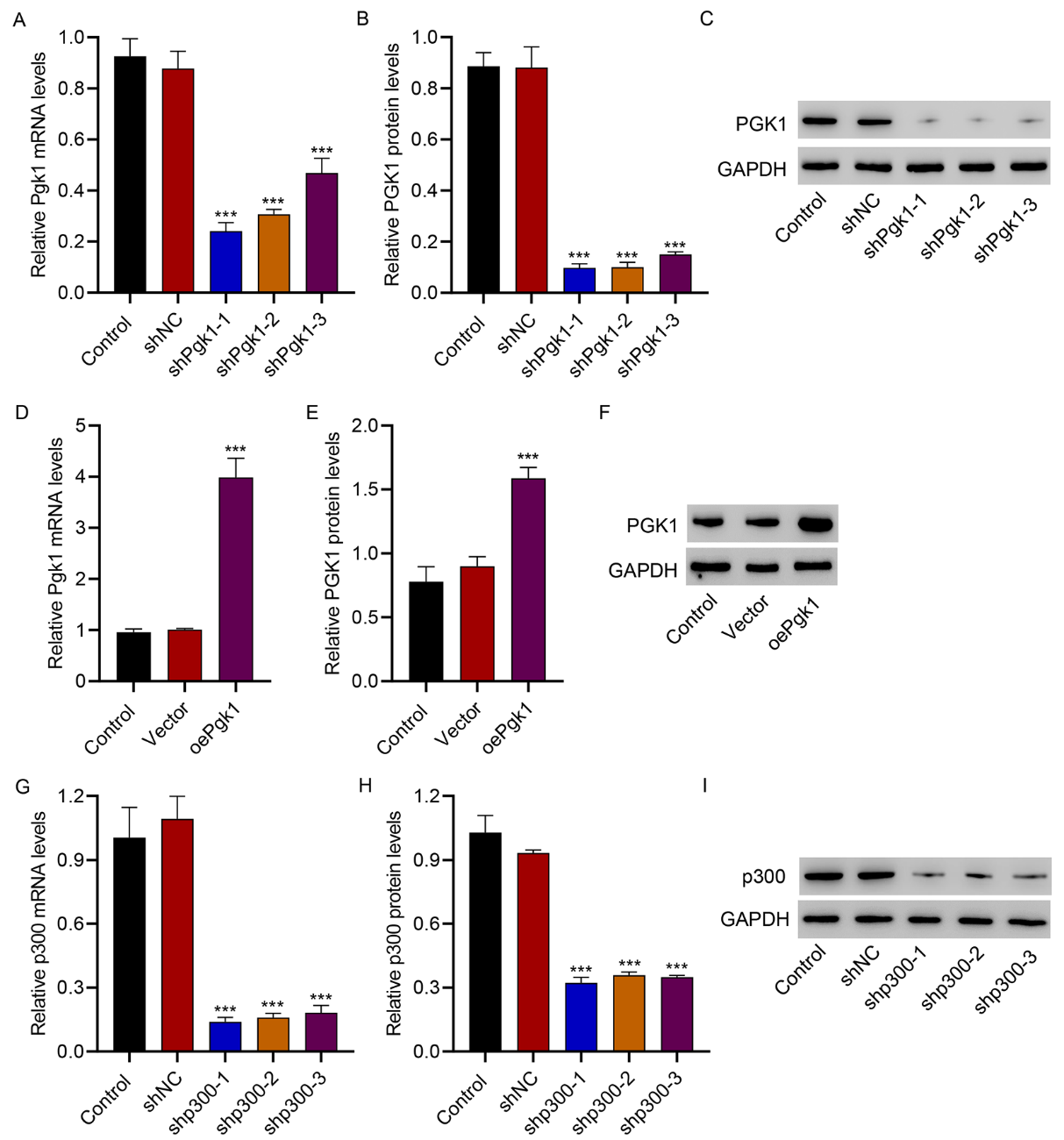


**Figure S1.** PGK1 and p300 expression in HAPI microglial cells. (A-C) Levels of PGK1 in HAPI cells transduced with PGK1 shRNA or shNC. (D-F) Levels of PGK1 in HAPI cells transduced with PGK1 overexpression plasmid or control vector. (G-I) Levels of p300 in HAPI cells transduced with p300 shRNA or shNC. *** p < 0.001 vs. shNC or vector.
